# A Review on Medicinal Properties of Orientin

**DOI:** 10.1155/2016/4104595

**Published:** 2016-05-19

**Authors:** Kit Ying Lam, Anna Pick Kiong Ling, Rhun Yian Koh, Ying Pei Wong, Yee How Say

**Affiliations:** ^1^School of Postgraduate Studies and Research, International Medical University, 57000 Kuala Lumpur, Malaysia; ^2^Division of Biomedical Science and Biotechnology, School of Health Sciences, International Medical University, 57000 Kuala Lumpur, Malaysia; ^3^Department of Biomedical Science, Faculty of Science, Universiti Tunku Abdul Rahman, Perak Campus, 31900 Kampar, Perak, Malaysia

## Abstract

Medicinal plants continue to play an important role in modern medications and healthcare as consumers generally believe that most of them cause fewer or milder adverse effects than the conventional modern medicines. In order to use the plants as a source of medicinal agents, the bioactive compounds are usually extracted from plants. Therefore, the extraction of bioactive compounds from medicinal plants is a crucial step in producing plant-derived drugs. One of the bioactive compounds isolable from medicinal plants, orientin, is often used in various bioactivity studies due to its extensive beneficial properties. The extraction of orientin in different medicinal plants and its medicinal properties, which include antioxidant, antiaging, antiviral, antibacterial, anti-inflammation, vasodilatation and cardioprotective, radiation protective, neuroprotective, antidepressant-like, antiadipogenesis, and antinociceptive effects, are discussed in detail in this review.

## 1. Introduction

Plants have been the key source of medicinal agents since ancient times. Based on fossil records, the use of medicinal plants most likely began in the middle Paleolithic about 60,000 years ago [1]. Over the years, traditional medicinal plants have been widely used to treat and/or to prevent diseases and even for daily consumption. For example,* Camellia sinensis* is an evergreen tree whose leaves and leaf buds are used to produce tea. The hot water extract of the dried leaf is taken orally as an antihypertensive tea in China, but to increase milk production for nursing mothers in Mexico. Indians also use the powder or decoction of dried leaves on teeth to prevent tooth decay [2]. The other example is* Gingko biloba*, which has traditionally been used to improve memory. The Chinese take the oil from fruit pulp to treat pulmonary tuberculosis but consume the hot water extract of the leaf orally as a vermifuge and also to treat asthma and senility [3]. Besides that, Koreans use the hot water extract of the* Gingko biloba* seed to induce labour or as an abortifacient [3]. In addition, the* Ocimum sanctum *Linn. (holy basil) has been used for the treatment of malaria, dysentery, skin disease, arthritis, bronchitis, asthma, and verminosis in the ancient Indian system of medicine [4, 5].

Traditionally, all of or part of a therapeutic plant such as garlic and cranberry is used as an herbal remedy. However, this has evolved in the recent drug discovery processes, in which bioactive compounds were isolated from plants and used as drugs, either in its original or in semisynthetic form [6]. Normally, semisynthesis of novel bioactive compounds with higher therapeutic values and lower toxicity can be done by modifying the structure of the existing bioactive compounds [7]. With this development, medicinal plants, particularly those with less toxic secondary metabolites, continue to play an important role in the modern medication and healthcare. According to Prasad and Tyagi, about 61% of 877 small-molecule drugs introduced worldwide between 1981 and 2002 were derived from natural products [8]. Some famous examples of plant-derived modern medicines are the cardiotonic drug (Digitoxin and Digoxin from* Digitalis purpurea *L.) [6–8], the anti-inflammatory drug (Aescin from* Aesculus hippocastanum *L.) [7], antitumour agent (Etoposide from* Podophyllum peltatum *L. [7, 8], Vincristine and Vinblastine from* Catharanthus roseus* [6, 8], Irinotecan and Topotecan from* Camptotheca acuminate* [8], Paclitaxel and Abraxane from* Taxus brevifolia* [8], Solamargine from* Solanum dulcamara* [8], Masoprocol from* Larrea tridentata* [8], Alitretinoin from* Daucus carota* [8]), antimalaria and antiarrhythmic agent (quinine and quinidine from* Cinchona *spp.) [6], muscle relaxant (atropine from* Atropa belladonna*) [6], and antinociceptive and cough suppressant drugs (morphine and codeine, resp.) from* Papaver somniferum* [6].

Considering the importance of bioactive compounds, extraction of these bioactive compounds from medicinal plants is a crucial step in producing plant-derived drugs. One of the bioactive compounds isolable from medicinal plants, orientin, is often used in the studies due to its extensive therapeutic properties. Orientin is a water-soluble flavonoid* C*-glycoside, which is commonly extractable from some medicinal plants such as* Ocimum sanctum* (holy basil) [9–11],* Phyllostachys nigra* (bamboo leaves) [12–18],* Passiflora* species (passion flower) [19, 20],* Trollius* species (Golden Queen) [21–24],* Jatropha gossypifolia* (Bellyache Bush) [25–28],* Linum usitatissimum* (flax) [29],* Commelina communis* (dayflower) [30],* Euterpe oleracea* Mart. (Acai palm) [31],* Ascarina lucida* [32],* Celtis africana* (white stinkwood) [33],* Croton zambesicus* (Lavender croton) leaves [34],* Cajanus cajan* (Pigeon pea) leaves [35], and* Thlaspi arvense* (Field Pennycress) [36]. The extraction of orientin in different medicinal plants and its medicinal properties, which include antioxidant, antiaging, antiviral, antibacterial, anti-inflammation, vasodilatation and cardioprotective, antiadipogenesis, antinociceptive, radiation protective, neuroprotective, and antidepressant-like effects, are discussed in this review.

## 2. Orientin

**Figure 1:**
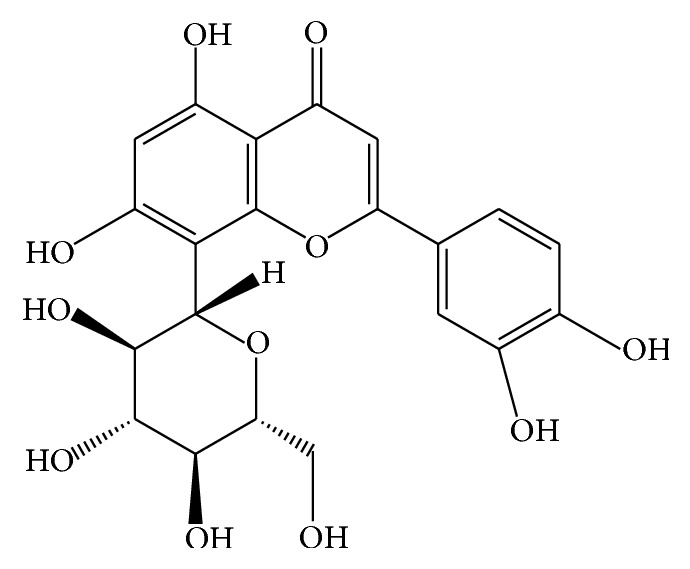
Chemical structure of orientin.

Orientin is a water-soluble flavonoid* C*-glycoside which has the IUPAC name of 2-(3,4-dihydroxyphenyl)-5,7-dihydroxy-8-[(2S,3R,4R,5S,6R)-3,4,5-trihydroxy-6-(hydroxymethyl)oxan-2-yl]chromen-4-one. It has a molecular formula of C_21_H_20_O_11_ and a molecular weight of 448.3769 g/mol [38]. The chemical structure of orientin (Figure 1) shows that it consists of mostly phenol groups with two ether groups and one ketone group. Therefore, a polar solvent such as methanol, ethanol, or water is required to extract orientin from the medicinal plants.

## 3. From Plants to Orientin

Orientin has been isolated from various medicinal plants such as* Ocimum sanctum, Phyllostachys* species (bamboo leaves),* Passiflora* species (passion flowers),* Trollius* species (Golden Queen), and* Jatropha gossypifolia* (Bellyache Bush).

*Ocimum sanctum* (also known as Indian holy basil or Tulsi in Sanskrit) was planted in India and it is well known for its medicinal values. The Ayurveda (Indian ancient system of medicines) uses different parts of* Ocimum sanctum* to treat cough, common cold, malarial fever, skin diseases, and snake bites [39]. Nair et al. successfully isolated orientin from the leaves of* O. sanctum *alongside with other flavonoids such as apigenin, apigenin-7-*O*-glucuronide, molludistin, luteolin, and luteolin-7-*O*-glucuronide [10]. Similarly, Uma Devi et al. isolated orientin from the same plant parts for* in vivo *radioprotective effect studies [11].

Bamboo leaves have been an essential resource in China for years and* Phyllostachys pubescens* Mazel ex H. de Lehaie is the most abundant species among the bamboo leaves in China due to its high yield, high growth rate, and extensive use [12]. Yong et al. isolated orientin and other compounds such as isoorientin, vitexin, isovitexin, caffeic acid, and ferulic acid by immersing different cultivars of* P. pubescens* in 70% methanol [16]. Besides that, Zhang et al. have isolated 49 mg of orientin from the antioxidant of bamboo leaves (AOB) concentrated solution initially from the 6.5 g of crude column chromatography fraction [17]. In addition, orientin has been separated from the three bamboo species:* Phyllostachys pubescens* Mazel ex H. de Lehaie,* Phyllostachys glauca* McClure, and* Pleioblastus yixingensis* by Sun et al. with newly proposed high-performance thin-layer chromatography (HPTLC) method [18]. With this simple method, 6.44, 7.82, and 4.92% (w/w) of orientin were produced from* P. pubescens*,* P. glauca, and P. yixingensis,* respectively [18].

Orientin has also been isolated from the* Passiflora *species.* Passiflora* was originally named in Spanish meaning “the flower with the five wounds” in the 17th Century by Spanish priests in South America. Up to now, there are about 400* Passiflora* species that have been recognised. It was suggested that* Passiflora incarnata* is used as a mild calming agent while other species were reported to possess medicinal properties [19]. In isolating orientin, Grundmann et al. managed to obtain 3.36 mg of orientin per gram of dried* P. incarnata* [19] while de Paris et al. isolated orientin from water extract of* P. edulis* dried leaves [20].

In Traditional Chinese Medicine,* Trollius chinensis* Bunge has been used to treat pharyngitis, respiratory infections, bronchitis, and tonsillitis for years. The dried flowers of other* Trollius *species were also discovered to possess various medicinal properties [37]. Liu et al. used HPLC method to isolate orientin from* Trollius chinensis* with the detection wavelength of 340 nm [21]. Besides that, 5 mg of orientin has been eluted from 15 kg of dried flowers of* Trollius ledebouri* [22]. In addition, Zhou et al. successfully isolated 95.8 mg of orientin from 500 mg of the crude extract of* Trollius ledebouri* with high-speed countercurrent chromatography (HSCCC) and semipreparative HPLC methods [23]. An ultraperformance liquid chromatography-electrospray ionization tandem mass spectrometric (UPLC-ESI-MS/MS) method has been developed by Li et al. in order to study the active compounds in* Trollius ledebouri* flower qualitatively and quantitatively. It was found that the smallest quantity of orientin that could be measured by this method was 515 *μ*g per gram of plant material [24].

*Jatropha gossypifolia* from Euphorbiaceae family is a tall shrub with plentiful fascicled bristles, originally from Brazil, but now can be found in all parts of India. The different parts of the plant are believed to have different medicinal values. For instance, the leaves are used as an insecticidal and wounds or ulcer healing agent, and the roots together with the leaves are used to treat anaemia, dysentery, fistula, and biliousness, while the seeds are used as aphrodisiacs, laxatives, and anthelmintic agents [40]. The seed oil is also a traditional medication for skin diseases such as itch, eczema, and herpes [40]. Orientin was isolated from the dried leaves of* J. gossypifolia* with the retention factor of 0.52 in TLC analysis [25–27]. Besides that, Pilon et al. used the crude leaf extracts of* J. gossypifolia* to improve the chromatographic resolution of HPLC-UV-diode-array detector (DAD) experiments to resolve the overlapping problem of chromatographic bands in HPLC between orientin and its isomer, homorientin [28].

Apart from the few plants mentioned above, there are some other medicinal plants that also contain orientin and yet are not as well studied. Orientin in the leaves and stems of* Linum usitatissimum *(flax) was first isolated together with some other compounds such as isoorientin, vitexin, isovitexin, vicenin-1, and vicenin-2 [29]. The natural herb that is used to treat type 2 diabetes,* Commelina communis* (dayflower), was found to contain orientin as well. The* in vitro* studies show that orientin has been one of the major antioxidants in this plant [30]. In addition, Gallori et al. have identified the presence of orientin in* Euterpe oleracea* Mart. (Acai palm) fruit by using HPLC-DAD-UV-Vis and HPLC-MS analysis [31]. Soltis and Bohm also successfully obtained orientin and some other flavonoids from the methanol extract of* Ascarina lucida* leaves [32]. Besides that, orientin was also isolated from* Celtis africana* (white stinkwood) [33],* Croton zambesicus* (Lavender croton) leaves [34],* Cajanus cajan* (Pigeon pea) leaves [35], and* Thlaspi arvense *(Field Pennycress) [36].

Table 1 summarises the extraction methods and amount of orientin extracted in each main medicinal plant discussed above.

## 4. Medicinal Properties of Orientin

### 4.1. Antioxidant and Antiaging

Orientin has been widely studied for its antioxidant property. The antioxidant property of orientin can be explained through the study of its electron affinity, electronegativity, electrophilic index, and adiabatic ionization potential of its high radical scavenging activity which is the ability to donate electrons [41]. The effect of orientin on hydrogen peroxide- (H_2_O_2_-) induced *β*-galactosidase activity also shows the antioxidant property of orientin. Orientin reduced the H_2_O_2_-induced *β*-galactosidase activity when compared to the increase of enzyme activity in H_2_O_2_ treatment alone [42]. Besides that, orientin significantly enhanced the survival of* Escherichia coli* mutants, DSH56 and DSH19 [42]. Nayak et al. also stated that the treatment of orientin highly reduced the mortality of the mutant cells, which is due to oxidation of macromolecules [42].

The antioxidant activity of orientin has also been shown in animal models. The D-galactose-treated aged mice were administrated with orientin for 8 weeks. An et al. found that orientin significantly increased the mice brain weights and general health status. The orientin-treated age mice also have increased levels of catalase, glutathione peroxidase, superoxide dismutase in serum, brain, liver, and kidneys, while the levels of malondialdehyde (a biomarker of oxidative stress) in the brain, liver, and kidneys as well as the levels of lipofuscin (an age pigment) were significantly decreased [43]. In addition, orientin also improves the structure of neuronal cell injuries in D-galactose-aged mice [43].

### 4.2. Antiviral and Antibacterial

The antiviral and antibacterial activities of orientin are beneficial for the future antibiotics development. Lin et al. [44] and Li et al. [45] have demonstrated that orientin has moderate or potent antiviral activity against Para 3 virus. Besides that, the flavonoids mixture (orientin, rutin, quercetin, and kaempferol) at the maximum nontoxic dose of 0.048 *μ*g/mL fully inhibited Herpes Simplex Virus Type 2 (HSV-2) of different viral titre (1,10,100 TCID_50_) on Hep-2 cells [46]. This is a great breakthrough in the medical field as HSV infection has been known to be a life-threatening disease. Apart from antiviral effects, orientin also expresses antibacterial effects. The study shows that the combination of flavonoids from* Ocimum sanctum, *orientin and vicenin, synergistically inhibited the growth of* Escherichia coli*,* Staphylococcus aureus*,* Staphylococcus cohnii, Klebsiella pneumoniae,* and* Proteus*, while the individual flavonoids were found to be less effective than the combined flavonoids [47]. The total flavonoids of* Trollius chinensis* include orientin and vitexin also showed evidence of antibacterial [44].

### 4.3. Anti-Inflammatory

Inflammation is a biological defence mechanism of tissue to injury or infection. Inflammation is characterized into acute and chronic phase in which they are differentiated by the presence of neutrophilic and monocytic leucocytes during the acute phase, whereas there are incidences of macrophages and lymphocytes during the chronic phase [48, 49]. It is well studied that the development of atherosclerosis is strongly related to vascular inflammation; therefore, there is an urge to develop an anti-inflammation medication which benefits vascular inflammation [50]. The biomarkers of vascular inflammation, high mobility group box-1 (HMGB1) protein, and endothelial cell protein C receptor (EPCR) have been investigated after the treatment of orientin. Orientin has been shown to inhibit the HMGB1 level in lipopolysaccharide- (LPS-) induced umbilical vein endothelial cells (HUVECs) as well as the HMGB1-mediated cytoskeletal rearrangements [51]. In addition, in the human endothelial cell lines, orientin suppressed LPS-induced membrane disruption, migration of monocytes, expression of cell adhesion molecules (CAMs), and LPS-induced EPCR detaching [52]. Furthermore,* in vivo* assessments showed that orientin inhibited HMGB1-mediated and LPS-induced hyperpermeability, CLP-induced release of HMGB1 level, leukocyte migration, LPS-induced tumour necrosis factor-*α* (TNF-*α*) level, interleukin-6 (IL-6) level, nuclear factor-*κ*B (NF-*κ*B) level, extracellular regulated kinases (ERK) 1/2 level, and lethality of mice [52]. Besides that, diabetes mellitus is the main complication in the progression of atherosclerosis by vascular inflammation. Ku et al. have also found that pretreatment of orientin inhibited the high glucose-induced CAMs, reactive oxygen species (ROS), and NF-*κ*B levels in HUVECs and mice [53].

### 4.4. Vasodilatation and Cardioprotective Effect

Presently, one of the most frequent cardiovascular diseases is hypertension, which increases the risk factor for congestive heart failure. Nevertheless, the maximum efficacy of the antihypertensive drugs is only 60% and typically two or more drugs from the categories of diuretics, calcium channel blockers, angiotensin converting enzyme or receptor blockers, adrenergic drugs, and vasodilators have to be combined to attain high efficacy on patients [54]. Therefore, vasorelaxant is an essential medication for hypertensive patients to reduce their elevated blood pressure and to protect the patients from cardiovascular diseases [54, 55].

Orientin has been identified to have vasodilatation effects on removed thoracic aortic rings from the New Zealand rabbit. It was found that orientin with an IC_50_ value of 2.28 *μ*M and 7.27 *μ*M relaxed phenylephrine-induced contractions in the endothelium-intact and endothelium-isolated aortic rings, respectively [56]. The possible pathway that orientin acts as a vasorelaxant on thoracic aortic rings is by the nitric-oxide-cGMP pathway, while in the vascular smooth muscle, it relaxes the muscle via activation of voltage-dependent calcium channels [56].

Besides that, orientin has been massively studied for its* in vivo* cardioprotective effect. Orientin was demonstrated to reduce myocardium apoptosis of rat heart with ischemia-reperfusion. The apoptosis of rat cardiomyocytes that were injured by hypoxia/reoxygenation also decreased upon pretreatment with orientin at 3, 10, and 30 *μ*mol L^−1^ [57]. Studies also showed that the protein levels of Bax, cytochrome c, and caspase-3 were reduced, whereas the level of bcl-2 was increased. This shows that orientin has antiapoptotic effect on ischemia-reperfusion and hypoxia/reoxygenation-treated heart and cardiomyocytes by deactivating the cytochrome c-caspase-3 mitochondrial apoptotic pathway [57]. On the other hand, Liu et al. showed that the myocardial structures, ventricular remodeling, electrocardiogram, and hemodynamic index of myocardial infarction rats have been improved after the treatment of orientin [58].

In addition, the cardioprotective effect of orientin has also been shown in dogs. Orientin increased endogenous antioxidase activity and reduced the oxygen-free radical formation by inhibiting the serum creatine phosphokinase and lactic dehydrogenase activities as well as enhancing superoxide dismutase activity in acute myocardial infarction dogs while reducing the myocardial infarction sizes [59]. In addition, the cardiac function upon orientin treatment has been observed in anesthetized open-chest dogs. Results showed that orientin was able to diminish maximal rising or dropping rate of intraventricular pressure (±*dp*/*dt*_max_) and left ventricular end dilation pressure and myocardial oxygen consumption but enhance cardiac output and coronary blood flow to boost up cardiac function in anesthetized dogs [60]. Moreover, orientin has been revealed to reduce arachidonic acid-induced blood platelet aggregation in rabbits and improve coronal flow in the removed heart of guinea pigs [61]. However, the* in vitro* study on the cardioprotective effect of orientin demonstrated the repolarization of mitochondrial membrane potential, reduced generation of reactive oxygen species, inhibition of mitochondrial cytochrome c, and the association with the PI3K/Akt signaling pathway by orientin on the H9c2 cardiomyocytes cells [62].

### 4.5. Radioprotective Effect

The radiation protective compounds have been widely studied recently due to the highly damaging power of ionizing radiation on human tissues. The damage of DNA and proteins by radiation can result in apoptosis, necrosis, mitotic death, or cell cycle interruption on the normal tissues [63]. Therefore, the search of radioprotective compounds is vital to protect human against radiation. Two flavonoids isolated from the leaves of* Ocimum sanctum*, orientin and vicenin, have been frequently examined for their radioprotective effect. The pretreatment of optimal dose at 50 *μ*g/kg body weight of orientin or vicenin 30 minutes on mice before being exposed to 11 Gy of gamma radiation showed protection against fatality, but the posttreatment of orientin or vicenin was not as effective as pretreatment [11]. The possible mechanisms of action of this protective effect were then determined. Both flavonoids have given protection against whole-body 3 Gy of gamma radiation-induced lipid peroxidation in mouse liver and also scavenged free radical activities include diphenylpicrylhydrazyl (DPPH) and 2,2′-azino-bis(3-ethylbenzothiazoline-6-sulfonic acid) (ABTS)* in vitro *[64]. Therefore, the antioxidant activity of the flavonoids might be the possible pathway of the radiation protective effect.

Additionally, in the Micronucleus (MN) assay, the treatment of orientin or vicenin at the optimum concentration of 17.5 *μ*M decreased the radiation-induced MN frequency, which indicates reduced chromosome damage in the human peripheral lymphocytes [65]. Besides that, the radiation protective effect of orientin and vicenin was demonstrated in mouse bone marrow as well. The intraperitoneal injection of orientin or vicenin significantly diminished the radiation-induced chromosomal aberrant cells and enhanced the number of exogenous spleen colonies (CFU-S) [66]. The* ex vivo* irradiated mouse splenocytes also showed a faster repairing effect and less radiation-induced DNA damage by orientin or vicenin [67]. The clonogenic survival of repair proficient cells was also elevated [9]. Furthermore, the combination treatment of orientin and vicenin together with two synthetic compounds WR-2721 and 2-mercaptopropionyl glycine (MPG) demonstrated reduced chromosomal aberration cells in bone marrow and significant drop in the percentage of aberrant metaphases [67]. These studies propose the potential of orientin and vicenin in protecting the normal tissues during the radiotherapy in cancer patients and the protection of radiation-related job workers such as radiologists.

### 4.6. Neuroprotective or Antidepressant-Like Effect

Neurodegenerative diseases are the incurable and weakening conditions where there are aggregation and deposition of misfolded intracellular and extracellular proteins, which lead to progressive central nervous system diseases [68, 69]. They have been emphasised among aging diseases because of the inadequate effective treatment, incurability, and associated economic and social burdens [70]. Therefore, there is an urge for the development of preventive or curative neuroprotective medications for the patients. A previous study using 3-(4,5-dimethylthiazol-2-yl)-2,5-diphenyltetrazolium bromide (MTT) assay reported that orientin at the concentration of less than 20 *μ*M was not cytotoxic to SH-SY5Y neuroblastoma cells [71]. In this study, the percentage of apoptotic cells was significantly decreased compared to the cells treated with 150 *μ*M H_2_O_2_ alone. Law et al. concluded that this antiapoptotic effect of orientin could have been attributed to the inactivation of caspases 3/7 and caspase-9 activities based on the caspase assays [71].

The neuroprotective effect has also been demonstrated in Amyloid *β*-Protein- (A*β*_1–42_-) induced mitochondrial dysfunction oxidative-stressed (Alzheimer's disease, AD) mice. Orientin was found to improve cognitive impairment of the AD mice and significantly reduced the levels of the oxidative stress biomarkers: 3-nitrotyrosine (3-NT), 4-hydroxynonenal (4-HNE), 8-hydroxy-2′-deoxyguanosine (8-OHdG), and ROS [72]. The mitochondrial apoptotic pathway was attenuated by reducing mitochondrial dysfunction by orientin. The possible pathway that orientin protects AD mice was found to be Nrf2/HO-1 redox signaling pathway as the expression of HO-1 was increased during the study [72].

Besides that, orientin also showed antidepressant-like effect. The chronic unpredictable mild stress (CUMS) mice showed enhanced central oxidative stress, amplified serotonin, and norepinephrine levels as well as less CUMS-induced depression-like behaviours upon oral treatment of orientin for 3 weeks [73].

### 4.7. Antiadipogenesis Effects

Adipogenesis is the process by which undifferentiated precursor cells differentiate into fat cells and further leads to the increasing prevalence of obesity among the society. Orientin together with some other flavonoids has been reported to exhibit potent antiadipogenesis activity. The orientin isolated from the ethanol extract of* Spirodela polyrhiza* exerts antiadipogenesis on lipid accumulation in 3T3-L1 cells [74]. In this study, Kim et al. discovered that the butanol soluble fraction had the highest adipogenesis and intracellular triglyceride accumulation inhibitory effect, by inhibiting the protein expressions of C/EBP*α* and PPAR*γ* [74]. C/EBP*α* and PPAR*γ* are the essential transcription factors during adipogenesis and participate in a single pathway of fat cell development with PPAR being the proximal effector of adipogenesis [75]. Considering the antiadipogenesis mechanism of orientin, it could be one of the useful therapeutic agents for obesity and type 2 diabetes patients.

### 4.8. Antinociceptive Effects

Pain is one of the most frequently observed symptoms of different pathologies. The principal targets of effective pain management are to ameliorate nociception, to reduce the threshold of pain sensation, and to improve quality of life [76]. The pain relieved effect of orientin has been demonstrated in models of pain in mice. Orientin with the ID_50_ of 6.5 mg/kg was able to reduce acetic acid-induced writhing and capsaicin- and glutamate-induced pain in mice [77]. Surprisingly, Da Silva et al. also discovered that orientin was 20-fold more potent than the typical painkiller, acetylsalicylic acid (aspirin), and 3.5-fold more dynamic than the common anti-inflammatory drug, indomethacin [77]. Thus, orientin can be an alternative antinociceptive treatment to the patients.

## 5. Concluding Remarks

The extensive studies of orientin on the medicinal properties of antioxidant, antiaging, antiviral, anti-inflammation, vasodilatation and cardioprotective, radioprotective, neuroprotective, antiadipogenesis, and antinociceptive effects can lead to promising therapeutic effect of orientin in the medical field. Yet the underlying mechanisms of these therapeutic properties are not well studied and remain indecisive. Besides that, orientin was found to have difficulty in crossing the blood-brain-barrier due to its hydrophilicity [78]. In addition, at this stage, most of the biological studies of orientin comprise only* in vitro* and preclinical investigations. Clinical data are yet to be available to support the use of orientin in patients. Therefore, the future research on orientin should be focused on the underlying pathways, the pharmacokinetics, and the tissue distribution in the human body in order to develop a highly effective drug which causes less adverse effects to patients.

## Figures and Tables

**Table 1:** Isolation of orientin from different medicinal plants.

Medicinal plants	Solvents	Separation and analysis methods	Amount of orientin extracted	References
*Ocimum sanctum*	Water	(i) Chromatography (ii) NMR	—	[10–12]

*Phyllostachys* species	Methanol or ethanol	(i) Chromatography (silica gel column or AB-8 resin column) (ii) HPLC (iii) HPTLC	(i) 49 mg of orientin from the AOB concentrated solution (ii) 6.44%, 7.82%, and 4.92% (w/w) of orientin were produced from *P. pubescens*, *P. glauca, and P. yixingensis,* respectively, by HPTLC	[15–19]

*Passiflora* species	Ethanol or water	(i) TLC (ii) HPLC	(i) 3.36 mg of orientin per gram of dried *P. incarnata* extract	[21, 37]

*Trollius* species	Ethanol	(i) HPLC (ii) Column chromatography (iii) HSCCC (iv) UPLC-ESI-MS/MS	(i) 5 mg of orientin from 15 kg of *T. ledebouri* dried flowers (ii) 95.8 mg of orientin from 500 mg of the crude extract of *T. ledebouri* (iii) Able to measure minimum 515 *μ*g of orientin per gram of plant material by UPLC-ESI-MS/MS method	[25–27]

*Jatropha gossypifolia*	Water, dichloromethane, ethyl acetate, *n*-butanol, or residual aqueous	(i) TLC (ii) HPLC	(i) The retention factor of orientin is 0.52 for TLC analysis (ii) Solve the problem of overlapping of the isomers (orientin and homorientin) chromatographic bands in HPLC	[30–33]

## Abbreviations

AOB: Antioxidant of bamboo leaves
HPLC: High-performance liquid chromatography
TLC: Thin-layer chromatography
UPLC-ESI-MS/MS: Ultraperformance liquid chromatography-electrospray ionization tandem mass spectrometric
HSCCC: High-speed countercurrent chromatography
H_2_O_2_: Hydrogen peroxide
HSV-2: Herpes Simplex Virus Type 2
TCID_50_: Median tissue culture infective dose
HMGB1: Mobility group box-1
EPCR: Endothelial cell protein C receptor
LPS: Lipopolysaccharide
HUVECs: Umbilical vein endothelial cells
CAMs: Cell adhesion molecules
TNF-*α*: Tumour necrosis factor-*α*
IL-6: Interleukin-6
NF-*κ*B: Nuclear factor-*κ*B
ERK: Extracellular regulated kinases
ROS: Reactive oxygen species
IC_50_: Half maximal inhibitory concentration
DPPH: Diphenylpicrylhydrazyl
ABTS: 2,2′-azino-bis(3-ethylbenzothiazoline-6-sulfonic acid)
MN: Micronucleus
CFU-S: Exogenous spleen colony
MPG: 2-Mercaptopropionyl glycine
MTT: 3- (4, 5-Dimethylthiazol-2-yl)-2,5-diphenyltetrazolium bromide assay
A*β*: Amyloid *β*-Protein
CUMS: Chronic unpredictable mild stress
ID_50_: Half maximal inhibitory dose.

## References

[1] Solecki R. (1975). Shanidar IV, a neanderthal flower burial in northern Iraq. *Science*.

[2] Ross I. A. (2005). *Medicinal Plants of the World, Volume 3: Chemical Constituents, Traditional and Modern Medicinal Uses*.

[3] Ross I. A. (2003). *Medicinal Plants of the World, Volume 1: Chemical Constituents, Traditional and Modern Medicinal Uses*.

[4] Pattanayak P., Behera P., Das D., Panda S. K. (2010). *Ocimum sanctum* Linn. A reservoir plant for therapeutic applications: an overview. *Pharmacognosy Reviews*.

[5] Das S. K., Vasudevan D. M. (2006). Tulsi: the Indian holy power plant. *Indian Journal of Natural Products and Resources*.

[6] Rates S. M. K. (2001). Plants as source of drugs. *Toxicon*.

[7] Fabricant D. S., Farnsworth N. R. (2001). The value of plants used in traditional medicine for drug discovery. *Environmental Health Perspectives*.

[8] Prasad S., Tyagi A. K. (2015). Traditional medicine: the goldmine for modern drugs. *Advanced Techniques in Biology & Medicine*.

[9] Satyamitra M., Mantena S., Nair C. K. K. (2014). The antioxidant flavonoids, orientin and vicenin enhance repair of radiation-induced damage. *Scholarena Journal of Pharmacy and Pharmacology*.

[10] Nair A. G. R., Gunasegaran R., Joshi S. (1982). Chemical investigation of some South Indian plants. *Indian Journal of Chemistry B*.

[11] Uma Devi P., Ganasoundari A., Rao B. S. S., Srinivasan K. K. (1999). *In Vivo* radioprotection by *Ocimum* flavonoids: survival of mice. *Radiation Research*.

[12] Zhang Y.-X., He C.-H. (2006). Process for extracting and separating total flavonoids from leaves of phyllostachys pubescens. *Journal of Chemical Engineering of Chinese Universities*.

[13] Lu B. Y., Wu X. Q., Shi J. Y., Dong Y. J., Zhang Y. (2006). Toxicology and safety of antioxidant of bamboo leaves. Part 2: developmental toxicity test in rats with antioxidant of bamboo leaves. *Food and Chemical Toxicology*.

[14] Sun W. X., Li X., Li N., Meng D. L. (2008). Chemical constituents of the extraction of bamboo leaves from *Phyllostachys nigra* (Loddex Lindl) Munro varhenonis (Mitf) Stepfex Rendle. *Journal of Shenyang Pharmaceutical University*.

[15] Xie J., Zhou P. P., Zhu X. Y., Liu X. J., Chen R. E., Wang P. (2010). Study on extraction of bamboo leaves flavonoids by homogenate extraction technique and its antioxidant activity. *Food Science and Technology*.

[16] Yong C. J., Hua L. L., Ke Y. (2011). Simultaneous determination of seven effective constituents in the leaves of bamboo by reversed phase high performance liquid chromatography (RP-HPLC). *Journal of Medicinal Plant Research*.

[17] Zhang Y., Jiao J., Liu C., Wu X., Zhang Y. (2008). Isolation and purification of four flavone *C*-glycosides from antioxidant of bamboo leaves by macroporous resin column chromatography and preparative high-performance liquid chromatography. *Food Chemistry*.

[18] Sun J., Yue Y., Tang F., Guo X. (2010). Simultaneous HPTLC analysis of flavonoids in the leaves of three different species of bamboo. *Journal of Planar Chromatography—Modern TLC*.

[19] Grundmann O., Wang J., McGregor G. P., Butterweck V. (2008). Anxiolytic activity of a phytochemically characterized *Passiflora incarnata* extract is mediated via the GABAergic system. *Planta Medica*.

[20] de Paris F., Petry R. D., Reginatto F. H. (2002). Pharmacochemical study of aqueous extracts of *Passiflora alata* Dryander and *Passiflora edulis* Sims. *Acta Farmaceutica Bonaerense*.

[21] Liu Z., Wang L., Li W., Huang Y., Xu Z.-C. (2004). Determination of orientin and vitexin in *Trollius chinesis* preparation by HPLC. *China Journal of Chinese Materia Medica*.

[22] Wu L.-Z., Wu H.-F., Xu X.-D., Yang J.-S. (2011). Two new flavone *C*-glycosides from *Trollius ledebouri*. *Chemical and Pharmaceutical Bulletin*.

[23] Zhou X., Peng J. Y., Fan G. R., Wu Y. T. (2005). Isolation and purification of flavonoid glycosides from *Trollius ledebouri* using high-speed counter-current chromatography by stepwise increasing the flow-rate of the mobile phase. *Journal of Chromatography A*.

[24] Li X., Xiong Z., Ying X., Cui L., Zhu W., Li F. (2006). A rapid ultra-performance liquid chromatography-electrospray ionization tandem mass spectrometric method for the qualitative and quantitative analysis of the constituents of the flower of *Trollius ledibouri* Reichb. *Analytica Chimica Acta*.

[25] Félix-Silva J., Souza T., Menezes Y. A. S. (2014). Aqueous leaf extract of *Jatropha gossypiifolia* L. (Euphorbiaceae) inhibits enzymatic and biological actions of *Bothrops jararaca* snake venom. *PLoS ONE*.

[26] Félix-Silva J., Souza T., Camara R. B. A. G. (2014). *In vitro* anticoagulant and antioxidant activities of *Jatropha gossypiifolia* L. (Euphorbiaceae) leaves aiming therapeutical applications. *BMC Complementary and Alternative Medicine*.

[27] Félix-Silva J., Gomes J. A. S., De Quadros Barbosa L. M. (2014). Systemic and local anti-inflammatory activity of aqueous leaf extract from *Jatropha gossypiifolia* L. (Euphorbiaceae). *International Journal of Pharmacy and Pharmaceutical Sciences*.

[28] Pilon A. C., Carneiro R. L., Carnevale Neto F. S., Bolzani V., Castro-Gamboa I. (2013). Interval multivariate curve resolution in the dereplication of HPLC-DAD data from *Jatropha gossypifolia*. *Phytochemical Analysis*.

[29] Dubois J., Mabry T. J. (1971). The *C*-glycosylflavonoids of flax, *Linum usitatissimum*. *Phytochemistry*.

[30] Shibano M., Kakutani K., Taniguchi M., Yasuda M., Baba K. (2008). Antioxidant constituents in the dayflower (*Commelina communis* L.) and their *α*-glucosidase-inhibitory activity. *Journal of Natural Medicines*.

[31] Gallori S., Bilia A. R., Bergonzi M. C., Barbosa W. L. R., Vincieri F. F. (2004). Polyphenolic constituents of fruit pulp of *Euterpe oleracea* Mart. (Açai palm). *Chromatographia*.

[32] Soltis D. E., Bohm B. A. (1982). Flavonoids of *Ascarina lucida*. *Journal of Natural Products*.

[33] Perveen S., El-Shafae A. M., Al-Taweel A. (2011). Antioxidant and urease inhibitory *C*-glycosylflavonoids from *Celtis africana*. *Journal of Asian Natural Products Research*.

[34] Wagner H., Hörhammer L., Kiraly I. C. (1970). Flavon-*c*-glykoside in *Croton zambezicus*. *Phytochemistry*.

[35] Pal D., Mishra P., Sachan N., Ghosh A. K. (2011). Biological activities and medicinal properties of *Cajanus cajan* (L) Millsp.. *Journal of Advanced Pharmaceutical Technology and Research*.

[36] Pang S., Ge Y., Wang L. S., Liu X., Lin C. W., Yang H. (2013). Isolation and purification of orientin and isovitexin from *Thlaspi arvense* linn. *Advanced Materials Research*.

[37] Wang R.-F., Yang X.-W., Ma C.-M. (2004). Trollioside, a new compound from the flowers of *Trollius chinensis*. *Journal of Asian Natural Products Research*.

[38] Koeppen B., Roux D. (1965). C-gylcosylflavonoids: the chemistry of orientin and iso-orientin. *Biochemical Journal*.

[39] Uma Devi P. (2001). Radioprotective, anticarcinogenic and antioxidant properties of the Indian Holy Basil, *Ocimum sanctum* (Tulasi). *Indian Journal of Experimental Biology*.

[40] Daniel M. (2006). *Medicinal Plants: Chemistry and Properties*.

[41] Praveena R., Sadasivam K., Deepha V., Sivakumar R. (2014). Antioxidant potential of orientin: a combined experimental and DFT approach. *Journal of Molecular Structure*.

[42] Nayak V., Nishioka H., Uma Devi P. (2006). Antioxidant and radioprotective effects of *Ocimum* flavonoids orientin and vicenin in *Escherichia coli*. *Defence Science Journal*.

[43] An F., Yang G. D., Tian J. M., Wang S. H. (2012). Antioxidant effects of the orientin and vitexin in *Trollius chinensis* Bunge in D-galactose-aged mice. *Neural Regeneration Research*.

[44] Lin Q. F., Feng S. Q., Cen Y. Z., Yang Y. T., Wang L. Y. (2004). Study on the antibacterial and antiviral activity compositions of *Trollium chinensis* Bunge. *Journal of Zhejiang University SCIENCE B*.

[45] Li Y.-L., Ma S.-C., Yang Y.-T., Ye S.-M., But P. P.-H. (2002). Antiviral activities of flavonoids and organic acid from *Trollius chinensis* Bunge. *Journal of Ethnopharmacology*.

[46] Boominathan S. P., Sarangan G., Srikakelapu S., Rajesh S., Duraipandian C., Srikanth P. (2014). Antiviral activity of bioassay guided fractionation of *Plumbago zeylanica* roots against Herpes Simplex Virus Type 2. *World Journal of Pharmaceutical Sciences*.

[47] Ali H., Dixit S. (2012). *In vitro* antimicrobial activity of flavanoids of *Ocimum sanctum* with synergistic effect of their combined form. *Asian Pacific Journal of Tropical Disease*.

[48] Feghali C. A., Wright T. M. (1997). Cytokines in acute and chronic inflammation. *Frontiers in Bioscience*.

[49] Gabay C. (2006). Interleukin-6 and chronic inflammation. *Arthritis Research and Therapy*.

[50] Brasier A. R., Recinos A., Eledrisi M. S., Runge M. S. (2002). Vascular inflammation and the renin-angiotensin system. *Arteriosclerosis, Thrombosis, and Vascular Biology*.

[51] Yoo H. Y., Ku S.-K., Lee T. H., Bae J.-S. (2014). Orientin inhibits HMGB1-induced inflammatory responses in HUVECs and in murine polymicrobial sepsis. *Inflammation*.

[52] Lee W. H., Ku S.-K., Bae J.-S. (2014). Vascular barrier protective effects of orientin and isoorientin in LPS-induced inflammation *in vitro* and *in vivo*. *Vascular Pharmacology*.

[53] Ku S.-K., Kwak S. Y., Bae J.-S. (2014). Orientin inhibits high glucose-induced vascular inflammation *in vitro* and *in vivo*. *Inflammation*.

[54] Sun T., Liu R., Cao Y.-X. (2011). Vasorelaxant and antihypertensive effects of formononetin through endothelium-dependent and -independent mechanisms. *Acta Pharmacologica Sinica*.

[55] Ogihara T., Matsuzaki M., Matsuoka H. (2005). The combination therapy of hypertension to prevent cardiovascular events (COPE) trial: rationale and design. *Hypertension Research*.

[56] Fu X.-C., Wang M.-W., Li S.-P., Zhang Y., Wang H.-L. (2005). Vasodilatation produced by orientin and its mechanism study. *Biological and Pharmaceutical Bulletin*.

[57] Fu X.-C., Wang M.-W., Li S.-P., Wang H.-L. (2006). Anti-apoptotic effect and the mechanism of orientin on ischaemic/reperfused myocardium. *Journal of Asian Natural Products Research*.

[58] Liu L. Y., Ma Q. Q., Li J. Y. (2013). The therapeutic effect of orientin on myocardial infarction rats. *Lishizhen Medicine and Materia Medica Research*.

[59] Fu X. C., Huang Z. X., Cai Y. W., Yang Q. H. (2006). Protective effects of orientin on experimental myocardial infarction in dogs. *Herald of Medicine*.

[60] Fu X. C., Yang Q. H., Huang Z. X., Li S. P. (2007). Effects of orientin on cardiac function and hemodynamics in anesthetized dogs. *West China Journal of Pharmaceutical Sciences*.

[61] Fu X.-C., Wang X., Zheng H., Ma L.-P. (2007). Protective effects of orientin on myocardial ischemia and hypoxia in animal models. *Journal of Southern Medical University*.

[62] Lu N., Sun Y., Zheng X. (2011). Orientin-induced cardioprotection against reperfusion is associated with attenuation of mitochondrial permeability transition. *Planta Medica*.

[63] Nair C. K. K., Parida D. K., Nomura T. (2001). Radioprotectors in radiotherapy. *Journal of Radiation Research*.

[64] Uma Devi P., Ganasoundari A., Vrinda B., Srinivasan K. K., Unnikrishnan M. K. (2000). Radiation protection by the *Ocimum* flavonoids orientin and vicenin: mechanisms of action. *Radiation Research*.

[65] Vrinda B., Uma Devi P. (2001). Radiation protection of human lymphocyte chromosomes *in vitro* by orientin and vicenin. *Mutation Research/Genetic Toxicology and Environmental Mutagenesis*.

[66] Nayak, Uma Devi P. (2005). Protection of mouse bone marrow against radiation-induced chromosome damage and stem cell death by the *Ocimum* flavonoids orientin and vicenin. *Radiation Research*.

[67] Uma Devi P., Bisht K. S., Vinitha M. (1998). A comparative study of radioprotection by *Ocimum* favonoids and synthetic aminothiol protectors in the mouse. *British Journal of Radiology*.

[68] Ross C. A., Poirier M. A. (2004). Protein aggregation and neurodegenerative disease. *Nature Medicine*.

[69] Skovronsky D. M., Lee V. M.-Y., Trojanowski J. Q. (2006). Neurodegenerative diseases: new concepts of pathogenesis and their therapeutic implications. *Annual Review of Pathology*.

[70] Hung C.-W., Chen Y.-C., Hsieh W.-L., Chiou S.-H., Kao C.-L. (2010). Ageing and neurodegenerative diseases. *Ageing Research Reviews*.

[71] Law B. N. T., Ling A. P. K., Koh R. Y., Chye S. M., Wong Y. P. (2014). Neuroprotective effects of orientin on hydrogen peroxide-induced apoptosis in SH-SY5Y cells. *Molecular Medicine Reports*.

[72] Yu L., Wang S., Chen X. (2015). Orientin alleviates cognitive deficits and oxidative stress in A*β*
_1–42_-induced mouse model of Alzheimer's disease. *Life Sciences*.

[73] Liu Y., Lan N., Ren J. (2015). Orientin improves depression-like behavior and BDNF in chronic stressed mice. *Molecular Nutrition & Food Research*.

[74] Kim J., Lee I., Seo J. (2010). Vitexin, orientin and other flavonoids from *Spirodela polyrhiza* inhibit adipogenesis in 3T3-L1 cells. *Phytotherapy Research*.

[75] Rosen E. D., Hsu C.-H., Wang X. (2002). C/EBP*α* induces adipogenesis through PPAR*γ*: a unified pathway. *Genes and Development*.

[76] Kilic F. S., Sirmagul B., Yildirim E., Oner S., Erol K. (2012). Antinociceptive effects of gabapentin and its mechanism of action in experimental animal studies. *Indian Journal of Medical Research*.

[77] Da Silva R. Z., Yunes R. A., De Souza M. M., Monache F. D., Cechinel-Filho V. (2010). Antinociceptive properties of conocarpan and orientin obtained from *Piper solmsianum* C. DC. var. *solmsianum* (Piperaceae). *Journal of Natural Medicines*.

[78] Li D. Q., Wang Q., Yuan Z. F. (2008). Pharmacokinetics and tissue distribution study of orientin in rat by liquid chromatography. *Journal of Pharmaceutical and Biomedical Analysis*.

